# Do perceived social support mitigate the influence of infertility stigma on fertility quality of life?

**DOI:** 10.3389/fgwh.2025.1577951

**Published:** 2025-07-30

**Authors:** Saher Al Sabbah, Ansarullah Tantry, Lisa Bayliss-Pratt

**Affiliations:** ^1^ Department of Psychology, Fatima College of Health Sciences, Abu Dhabi, United Arab Emirates; ^2^ Department of Nursing, Fatima College of Health Sciences, Abu Dhabi, United Arab Emirates

**Keywords:** infertility, female's health, infertility stigma, fertility quality of life, perceived social support

## Abstract

**Introduction:**

Infertility is a medical condition that affects both males and females and can cause the individuals biopsychosocial, spiritual, and medical detriments. Quality of life among such couples or singles is a matter of concern. The question that we need to address is whether infertility affects the quality of life. Does the stigma associated with Infertility deter Infertile females from leading normal lives? This research explores how infertility stigma affects the quality of life of infertile females and whether perceived social support reduces the stress related to stigma thereby contributing to a better quality of life among females battling Infertility in India.

**Methods:**

Participants from Jammu and Kashmir who identified as currently or previously infertile discussed their feelings about fertility stigma, the quality of their fertility-related social support, and their fertility quality of life. Only 302 fully complete questionnaires were obtained from the 351 identified individuals who were given data collection tools. Structural Equation Modeling (SEM) was used to treat data.

**Results:**

It was seen that infertility stigma and perceived social support had an impact on fertility quality of life, either directly or indirectly. Infertility quality of life was reduced by stigma (*β* = -.413, SE = .017, *p* ≤ .01 level of significance, CI, 95%), and this link was partially mediated by infertile female's perceptions of social support (*β* = .512, *p* ≤ .01 level of significance, CI, 95%). In other words, it can be said that the negative effects of infertility stigma were buffered by perceived social support and improved fertility quality of life. Additionally, the sense of stigma was adversely linked with the overall quality of past fertility-related support.

**Discussion:**

The findings of study confirms that perceived social support significantly mitigates the negative impact of infertility-related stigma on fertility quality of life among infertile females, highlighting the crucial role of emotional and social resources in mitigating distress. These findings emphasize the importance of encouraging supportive environment and interventions to enhance quality of life in females experiencing infertility stigma.

## 1 Introduction

Infertility is the evident consequence, and fertility is a universal human species continuation concern (1). The global prevalence rate for 12-month infertility was reported by Barrera et al. to range from 3.5% to 16.7% in more developed countries and from 6.9% to 9.3% in less-developed nations (2). The prevalence of infertility in low-to-middle-income countries was prevalent in 31% (27.9%–34.7%) of females aged 18–44 years (3). Globally, 72.4 million females are infertile, of which 40.5 million seek medical help (2), with India accounting for between 15 and 20 million (25 percent) (4). The scope of the problem necessitates an immediate response, especially since most cases of infertility are preventable.

In India, 28 million couples struggle with infertility. However, only about 1% of people seek medical advice and treatment for this problem (5). According to WHO estimates the overall prevalence of primary infertility in India ranges from 3.9 percent to 16.8 percent (6). It is a syndrome created mostly by biological and environmental circumstances, with a tendency to internalize it as a personal or individual failing. Misplaced social views, such as blaming females primarily for conception or refusing to consider the male part in fertility, exacerbate the problem. This can make it difficult to talk honestly about the problem with others or seek therapy. In most societies, ‘not having children’ is an unwelcome social role, and infertility is a 'surprising life adjustment’ (6, 7). Infertile females in impoverished countries face an additional disadvantage: their ability to participate in societal activities is severely constrained (8). Affordability of therapy, lack of insurance coverage, absence of quality facilities in tier II and III geographies, and inconsistent treatment reached out to patients are all obstacles when it comes to battling infertility (9, 10). Despite the numerous social, psychological, economic, and medical consequences for both males and females, infertility is a stigmatized condition that is frequently overlooked as a major health concern (9).

Little is known about females who identify an infertility problem but do not seek treatment. Although the experience of infertility and involuntary childlessness is acknowledged as a serious life stressor marked by a loss of control, bodily integrity, and identity (11). Infertility stigma is a phenomenon linked to a variety of psychological and social stresses, particularly among females. The stigma is linked to feelings of humiliation and secrecy (12). Stigma is a negative sense of being different from others in society and of going against societal norms (13). If infertility is perceived as a stigma, it could isolate the infertile person from social support, leading to despair, anxiety, stress (14), guilt (15), and interpersonal issues (14, 16). It can also lead to psychological problems, such as low self-esteem and self-efficacy, as well as a proclivity for self-stigma (17). According to Taebi et al. (12) same-sex stigma is widespread, with most infertile female patients complaining of being stigmatized by other females.

Quality of life is the way that various facets of a person's life (e.g., physical and mental health, social and spiritual life) influence how they perceive their position in the world (18). The fact that most couples (60 percent) will not be able to conceive without medical assistance after just six months of attempting makes infertility special (19). Because of this, infertility is not an issue that can be resolved quickly; those who experience it may find it difficult to conceive for months or even years. The real well-being issue is typically a big part of an individual's identity for those diagnosed with infertility, and it can introduce additional factors that impair quality of life (20, 21). The quality-of-life scale, which is one of the most widely used and trustworthy indicators of people's quality of life with chronic illnesses, assesses social connections, general material, and physical well-being, participation in pursuits, self-improvement, and leisure (22), but it does not consider the impact of diseases on quality of life wholly. FertiQoL is a one-of-a-kind technique to measure the quality of life for those who are battling with infertility, while similar health-related quality-of-life measures are frequent in the literature.

Stigma is a social construct that emerges because of social interaction (23). It's a cyclical process that starts when people realize they're different from the folks they're around. This distinction has a negative meaning that causes the stigmatized individual to feel devalued, whether real or imagined. The perceived stigma is increased because of this sense of social devaluation, and the cycle of social judgment and comparison persists. People with stigmatized problems are more likely to suffer from depression (24), as well as mood and anxiety disorders (25). Because these mental health issues have a negative influence on the overall quality of life, stigma is a significant consideration for those who are trying to conceive. There are three ways to deal with stigma: passing, internalizing, or coping (26). Individuals who pass unless the stigmatizing condition is present and thus refuse to accept it are said to be passing. Internalized stigma occurs when people accept their stigmatizing condition but don't communicate about it; it's linked to physical health issues, such as chronic illness diagnosis (27).

### 1.1 Aim

The purpose of the study was to investigate the connection between Kashmiri females's fertility quality of life and the stigma associated with infertility. The study also sought to determine how perceived social support mediated the association between fertility quality of life and infertility stigmas.

### 1.2 Objectives

1. To study the relationship between infertility stigma and fertility quality of life among Kashmiri females.
2. To assess the mediating role of social support in buffering the negative impact of infertility stigma on fertility quality of life.
3. To provide empirical evidence on the psychosocial determinants of fertility-related quality of life in a culturally sensitive context.
4. To offer insights for developing social and psychological interventions aimed at improving the well-being of females facing infertility stigma.

## 2 Theoretical foundations: stress-buffering hypothesis

The Stress Buffering Hypothesis proposed by Cohen and Wills (28) provided an outstanding theoretical framework for this study, as it emphasized the role of social support in buffering the negative effects of stressors on an individual's outcome (e.g., well-being), so it has three components such as stressor, social support, and an outcome. The buffer hypothesis aligned with the present study's hypothetical framework that perceived social support mitigates the negative effects of infertility-related stigma on fertility quality of life among infertile females. The buffer hypothesis suggested that the effectiveness of social support depends on the presence of stressors. Social support has the most significant impact when individuals experience high levels of stress (28).

For infertile females experiencing stigma, social support is hypothesized to alleviate the emotional and social burden, thereby preserving their fertility quality of life. The hypothesis emphasized the importance of perceived social support, which refers to an individual's belief that support is available when needed.

In this study, infertile female's perception of social support might play a critical role in buffering the effects of infertility-related stigma on their fertility quality of life. Stigma is the environmental stressor that might be negatively affecting the fertility quality of life of infertile females. It leads to reduced self-esteem, isolation, and impaired quality of life (29). The hypothesis predicted that infertile females who receive strong social support will have better quality of life outcomes compared to those with less support, even in the presence of stigma. The hypothesis provided a well-defined explanation of how social support mediates the impact of stressors, which is central to this study.

**Figure 1:**
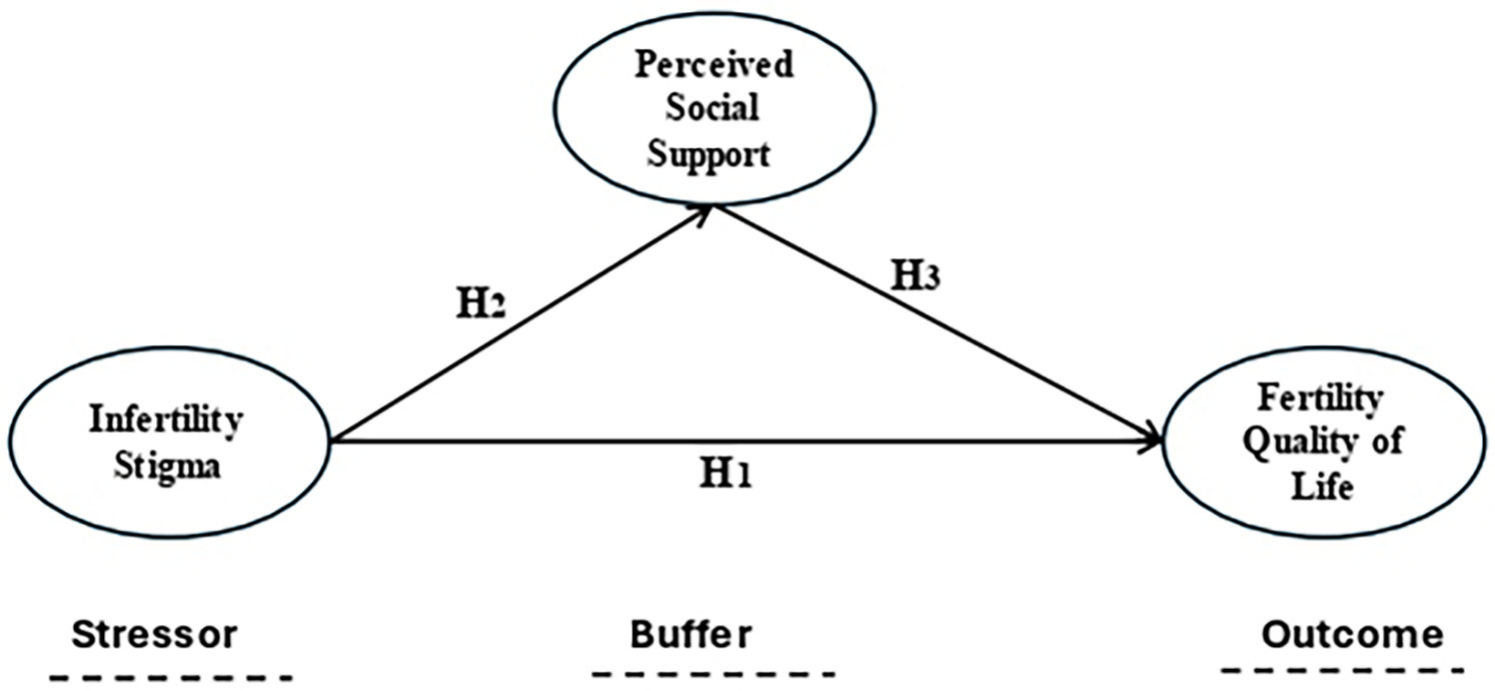
Theoretical foundations of the conceptual model of the study.

The findings of this study can highlight the importance of strengthening social support networks as part of interventions aimed at improving the fertility quality of life of infertile females. Additionally, public health programs could focus on educating families and social authorities about their role in buffering the effects of stigma and providing emotional and practical support. Therefore, the Stress Buffering Hypothesis was a highly relevant and robust theoretical framework for this study (see Figure 1).

### 2.1 Hypotheses

*H_1_:* Infertility stigma is negatively correlated to fertility quality of life among infertile females.
*H_2_:* Infertility stigma is negatively correlated to perceived social support among infertile females.
*H_3_:* Perceived social support mitigates the negative effects of infertility-related stigma on fertility quality of life among infertile females.

## 3 Methods

### 3.1 Design

The current descriptive study used quantitative research methods. Moreover, a cross-sectional correlation design was followed to attain the aim of the study.

### 3.2 Sample

A total of 351 females who reported being infertile at the time of the data collection or in the past took part in the study sample. The data collection tool was administered on the initially recruited 351 infertile females, however, 38 respondents did not respond back or responded incompletely and additional 11 responses were excluded on data cleaning. Therefore, the final sample remained of 302 respondents. The infertility status of a woman was considered if it was diagnosed by a doctor. Therefore, the participants included in the study were diagnosed by doctors. The participants for the present study included 302 infertile females from Jammu and Kashmir. The statistics of female infertility were collected from District hospitals and Major Gynecological clinics for sampling. As per the medical records, there were 3016 infertile females in the 25–55 years age group. Therefore, 10% of the identified sample, i.e., 302, was taken as a final sample that best matched the criteria. The age of the patients included in the sample ranged from 25 to 55 years. The average age of the participants was 34.23 years. Most participants were currently married (89.7%, *n* = 271), and 10.3% (*n* = 31) were divorced. Most sample participants had been given the infertile diagnosis by a doctor (77.15%, *n* = 233) while 22.84% had not (*n* = 69) but stated that they were unable to conceive and that they self-identified as infertile. Participants were enlisted using snowball sampling through clinical, social and personal networks after receiving consent from human subjects.

### 3.3 Materials

The scales used to collect data from the sample were FQoL Scale, Perceived Social Support Scale, and Infertility Stigma Scale.

The FQoL Scale was used to assess the quality of life specifically associated with infertility (22). The primary module that was utilized consists of 24 items measuring the quality of life associated with infertility. The 5-point Likert scale formats were used to collect responses for each subscale. Cronbach's alpha of the scale for the current study was.83.

The perceived social support Scale developed by Zimet, et al. (30) was employed for examining the perception of social support among infertile females in India. It has 12 items with 7-point ratings on all items. Cronbach's alpha of the scale for the current study was.81 which is above the acceptable range (31).

Infertility Stigma as measured on Infertility Stigma Scale constructed by Fu, et al. (32) The scale contained 27 items with responses on each item on a 5-point Likert type scale (1 = totally disagree, 2 = partially disagree, 3 = uncertain, 4 = partially agree, 5 = totally agree). The Cronbach's alpha of the scale was.94 (32). Cronbach's alpha of the scale for the current study was 87.

The reliability of the tools was tested for the current study by calculating Cronbach's alpha which was in the range of .81–.87, hence, satisfactory reliability coefficients (31). Scoring was done by adding responses for all the measures separately.

Therefore, the above scales were compiled into a questions booklet and administered to the identified sample.

### 3.4 Data analysis

The data were analyzed by using descriptive statistics of mean and standard deviation to make the data meaningful. Pearson's correlation was calculated to find the potential relationships between the variables. Furthermore, Structural Equation Modeling was used to examine the mediation impact of perceived social support on the relationship between Infertility stigma and fertiQoL using AMOS (V.24) (33).

### 3.5 Ethical consideration

Permission was granted from the Research Ethics Committee before approaching the participants for data collection. Also, proper consent was received from individual participants for voluntary participation in the study. The purpose of the study was disclosed to all the participants. The subjects were assured of the confidentiality of the information.

## 4 Results

The mean FertiQoL score is 80.81 with a standard deviation of 5.997, indicating moderate variability. Perceived social support (PSS) has a mean of 33.15 with a standard deviation of 8.497, showing relatively higher dispersion. Infertility stigma (IS) has the highest variability with a mean of 51.70 and a standard deviation of 19.918, and its skewness (1.014) suggests a slightly positively skewed distribution which is under acceptable range of normality of data. The total sample is 302 (Table 1).

**Table 1:** Descriptive statistics.

Variables	Mean	SD	Skewness	Kurtosis
FertiQoL	80.81	5.997	−.299	−.476
PSS	33.15	8.497	.050	−.091
IS	51.70	19.918	1.014	.714
Valid N	302			

Fertility quality of life (FertiQoL) is positively correlated with perceived social support (PSS) (*r* = .702, *p* = .001), indicating that higher social support is associated with better fertility-related quality of life. Conversely, infertility stigma (IS) shows a negative correlation with both FertiQoL (*r* = −.398, *p* = .001) and PSS (*r* = –.461, *p* = .001), suggesting that greater stigma is linked to lower quality of life and reduced social support. All correlations are statistically significant at *p* < .001 (Table 2).

**Table 2:** Inter-correlations matrix.

Variables	Statistics	FQOL	PSS	IS
FertiQoL	Pearson correlation	–	.702**	−.398**
	*P* value		.001	.001
PSS	Pearson correlation		–	−.461**
	*p* value			.001
IS	Pearson correlation			–
	*P* value			

** Significant at 0.01 level.

### 4.1 SEM results

The model fit of the measurement model was examined by first examining the model fit of all the variables in the study. The structural model fits indices were found to be good [*X*^2^/*df* = 203.180, TLI = .951, CFI = .957, RMSEA = .038, C.I, 95% (.042; .067), P-Close = .571], suggesting a good model fit (Table 3).

**Table 3:** Goodness of Fit: confirmatory factor analysis Fit indices for the model (*N* = 302).

Model	*χ*^2^/*df*	df	TLI	CFI	RMSEA	*P*-close
	203.180	71	.951	.957	.038	.571

*χ*^2^, Chi Square; *df*, degree of freedom; TLI, Tucker–Lewis's index; CFI, comparative fit index; RMSEA, root mean square error of approximation; *p*, probability value.

**Figure 2:**
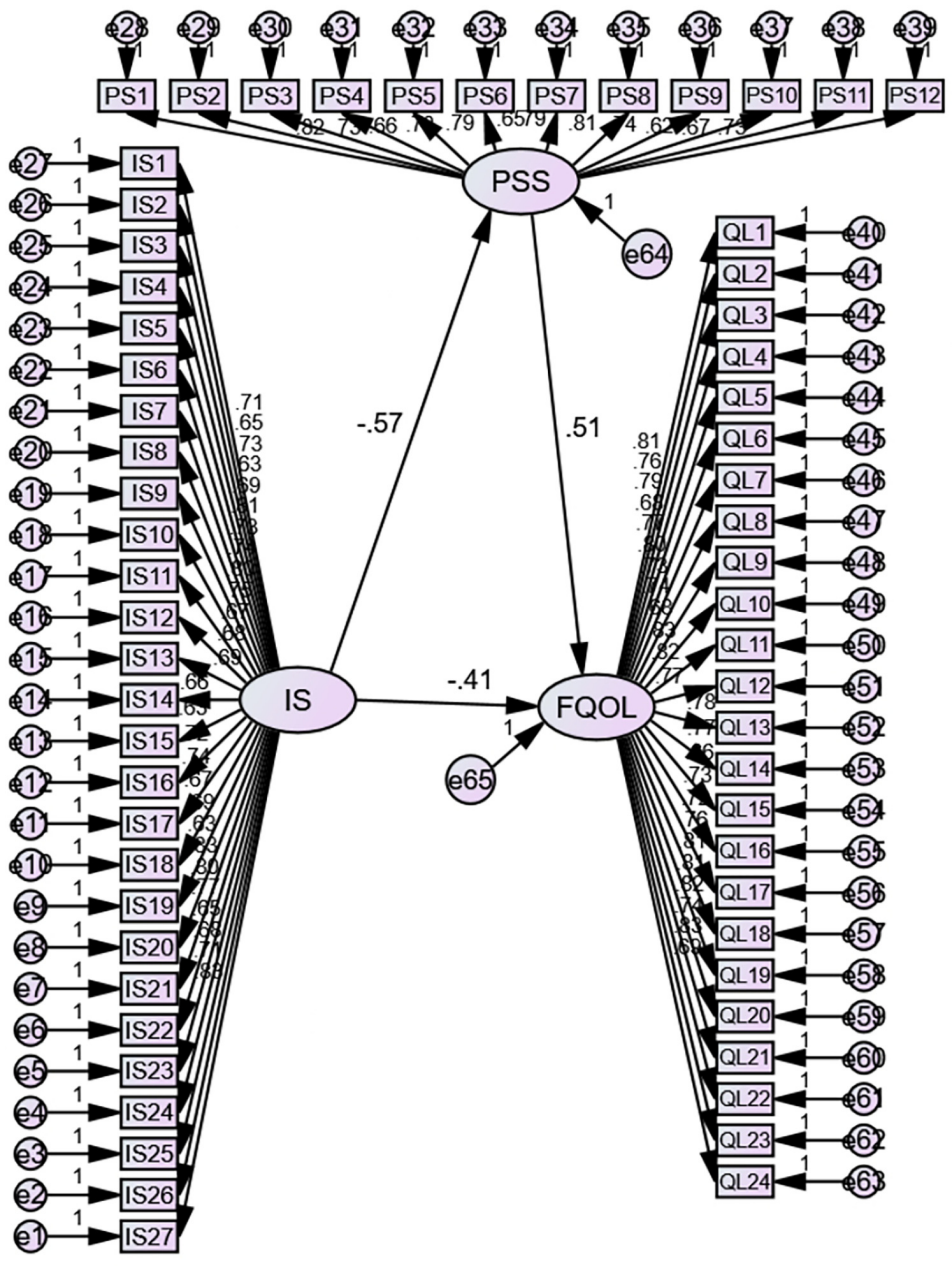
SEM model determining the relationship between infertility-related stigma (iS), perceived social support (PSS/PS), and fertility quality of life (FQOL/QL).

After the successful estimation of the measurement model with reasonable model fits, the structural model was used to test the hypotheses postulated in this study, the structural model permits researchers to test complex hypotheses simultaneously. Mediation results presented in Figure 2 offer support for all three hypotheses of the study. The findings of the structural path showed the total, direct and indirect effects of the model. Findings showed that fertility quality of life was influenced negatively by perceived stigma (*β* = −.413, SE = .017, *p* ≤ .01 level of significance, CI, 95%). Therefore, hypothesis 1 that infertility stigma is negatively correlated to fertility quality of life among infertile females is supported. The findings showed that stigma associated with infertility had a significant and meaningful negative impact on fertility quality of life, with a tendency for stigmatized females to have lower fertility quality of life. It was also found that infertility stigma is inversely related to perceived social support among infertile females (*β* = −.567, SE = .019, *p* ≤ .01 level of significance, CI, 95%). Therefore, supporting hypothesis 2 that infertility stigma is negatively correlated to perceived social support among infertile females. The findings are presented in Table 4.

**Table 4:** Estimates of direct and indirect effects.

Relationship	Direct effect	Indirect effect	Confidence interval		*p* value	Conclusion
			Lower bound	Upper bound		
IS → PSS → FQOL	.413 (.001)	.512	.156	.467	.001	Partial mediation

IS, infertility stigma; PSS, perceived social support; FQOL, fertility quality of life.

To test H_3_, using AMOS- structural equation modeling for path analysis was conducted which suggested that infertility stigma indirectly influenced infertile female's fertility quality of life through its effect on perceived social support. Specifically, stigma decreased fertility quality of life, and this link was partially buffered by the infertile females perceived social support (*β* = .512, *p* ≤ .01 level of significance, CI, 95%). In other words, it can be said that perceived social support reduces the magnitude of the impact of infertility stigma on fertility quality of life. Although there was also a direct effect of stigma on fertility quality of life, as shown in Figure 2, participants with more stigma who reported lower perceived social support and those with lower perceived social support also reported lower fertility quality of life. Thus, H_3_ which claimed that perceived social support mitigates the negative effects of infertility-related stigma on fertility quality of life among infertile females is supported.

## 5 Discussion

This study aimed to investigate the link of infertility stigma with fertility quality of life among Kashmiri (India) females. The study further examined the mitigating role of perceived social support between infertility stigmas with fertility quality of life. The first hypothesis (H_1_) of the study was that infertility stigma is negatively correlated to fertility quality of life among infertile females. The results supported the hypothesis and suggested that infertility-related stigma is inversely linked to fertility quality of life, such that females who endured greater stigma are also likely to have a lower quality of life for fertility. Previous research, such as Boudewyns et al. (34) revealed similar outcomes when looking into how people talked about a positive sexually transmitted illness diagnosis. According to their findings’, perceived stigma was adversely connected to the chance of having sexually transmitted illness talks with both sexual partners and social network members. They also discovered that disorders associated with increased stigma (such as chlamydia, gonorrhea, herpes, HIV, HPV, and syphilis) were reported on social media substantially less frequently. In conclusion, stigma is likely to reduce the likelihood of people talking about and obtaining help for their health problems. High and Steuber (35) looked at how various forms of disclosure (including direct disclosures, entrapment, indirect media, incremental disclosures, use of humor, and third-party member disclosures) affected how people felt about each other, their ability to conceive and have children, their quality of life in general, and their perception of social support. In conclusion, perceived social support appeared to mediate the association between disclosure technique, fertility quality of life, and overall quality of life. The level of social support completely moderates the association between disclosure strategy and fertility quality of life, implying that social support is crucial in figuring out how disclosure and fertility-related quality of life interact. Furthermore, female's quality of life improved when they thought social support related to fertility was effective.

The findings of our study supported prior research (24, 25, 36) which found that the sense of stigma reduces fertility quality of life for people who are presently or have previously struggled with infertility. Our findings specifically revealed a substantial detrimental connection between stigma perceived and fertility quality of life. However, these findings supported those of Abdullahzadeh et al. (37) who found that perceived stigma exacerbated infertility-related misery in both males and females, as measured by melancholy and anxiety. The findings of this study not only confirmed that emotional distress can develop because of infertility-related stigma but also that this suffering is not only temporary but can have a long-term negative influence on quality of life. Furthermore, these findings added to the existing stigma and infertility literature. As a result, it can be claimed that when infertile females in India felt stigmatized because of their infertility, their fertility quality of life suffered as well.

The results also supported the second hypothesis (H2), which holds that infertility stigma has a negative relationship with infertile female's perceptions of social support. As a result, the findings revealed that infertility stigma is negatively associated with infertile female's perceptions of social support. The load may be exacerbated by erroneous social views such as thinking females are primarily responsible for conception or a refusal to understand the male element in fertility. This can make it difficult to talk honestly about the problem with others or seek therapy. In most societies, not having children is an unwelcome social role, and infertility is a surprising life adjustment (11). Infertile females in impoverished countries face an additional disadvantage: their ability to participate in societal activities is severely constrained (9). Smith et al. (38), Jacobson (8), and Birtel et al. (39) demonstrated a negative connection between stigma and social support. However, this makes a significant contribution to the field of infertility research.

Infertility is a sudden change in one's life, and “not having children” is viewed as a bad social role in many countries (11). Another disadvantage for infertile females in developing nations is that their participation in social activities is severely limited (9). In studies of HIV stigma and social support, Smith et al. (38) and Zeligman et al. (40) discovered a negative association. However, it contributes significantly to the field of infertility research. The findings back up prior research, such as a study by High and Steuber (35) which revealed that fertility quality of life and overall quality of life is altered in distinct ways, which supports this study's reason for focusing on fertility quality of life. Their findings revealed that the relationship between direct disclosures and overall quality of life was mediated by perceived social support to some extent. As a result, if a stigmatized person was less likely to seek social assistance and more likely to feel shame, and social support they did receive could have been unsatisfying through the lens of their existing stigma. The findings also support the idea that social support workers might help people feel less stigmatized about their infertility. The sheer feeling of societal acceptance for both partners in an infertile marriage decreased the stigma, according to Boivin et al. (14) and Bornstein et al. (13). As a result, having a social network to whom you may reveal your ailment may reduce the stigma associated with it. As a result, members of social networks may have the greatest impact on perceived stigma during the early stages of diagnosis. Another study supported that the quality of life of infertile females lies in an irony of concerns to deal with, and they face so many challenges and exercise efforts to take the help of social support to cope with infertility-related concerns (21). On the other hand, it was found that males with infertility had a better quality of life than females with infertility, according to a study (37, 41, 42). Social support and coping mechanisms significantly buffer the negative effects of infertility-related stigma and stress, thereby enhancing fertility quality of life among both infertile females and males (43–48). These findings were supported by the findings of the present study, showing that the negative impact of infertility-related stigma on fertility quality of life of infertile females was buffered by perceived social support. Therefore, the findings supported the third hypothesis (H_3_) which postulated that perceived social support mitigates the negative effects of infertility-related stigma on fertility quality of life among infertile females.

### 5.1 Limitations

The findings of this research have several major ramifications. To begin with, stigma has a tremendous impact on one's quality of life, which is difficult to minimize without communication. This raises the question of how members of social networks and practitioners may assist infertile people. Focusing on how to train physicians and social connection members, how to provide good social support, and to coach them on what kind of messages reduce stigma perception. To do so, researchers will need to figure out what kinds of communications cause the recipient to feel stigmatized. One solution might be to talk to people on social media about the potentially harmful repercussions of asking newlyweds when they plan to have children. In conclusion, this study emphasizes the importance of tempering the normative cultural rhetoric that having children is expected and that any couple who deviates from this path is wrong. Furthermore, the results of this study suggest that social support reduces the impact of stigma. Perhaps researchers should analyze the disparity between the sorts of social support received and the types of assistance desired to further dissect the data. Along these grounds, it may be necessary to assess specific social support messages, infertile individuals’ perceptions of these signals, and the social support provider's overall goal. Scholars could discover how to most effectively teach individuals to provide social support to network members by coping with infertility by doing such a study, which could have a favorable impact on quality-of-life outcomes. Finally, this research sheds light on the impact of infertility in other Indian states, as well as other parts of the world.

The social and emotional effects of infertility on females must be understood by healthcare practitioners. It's critical to evaluate a woman's relationship with her spouse, her extended family, and her social support system to determine the extent of social isolation that can affect her general health and quality of life. Health care specialists must be sensitive to how infertility affects a woman's life, her reaction to following life events, and the significance she attaches to changes in life stage to effectively address the consequences of infertility.

### 5.2 Implications

The findings of this research have important implications for mental health practitioners, legislators, and support groups that assist infertile females in Kashmir in particular and other parts of the world in general. There is an urgent need for focused interventions to lessen societal stigma and raise awareness of infertility as a medical issue rather than a social failure, given the detrimental effects of stigma associated to infertility on quality of life. The study also emphasized the importance of perceived social support in reducing the negative impacts of stigma, stressing the necessity of peer support groups, counselling services, and community-based programs to improve support systems for impacted females. To improve coping strategies and general wellbeing, medical professionals should incorporate psychosocial assistance into infertility treatment regimens. Policymakers should also think about launching educational initiatives to dispel cultural myths and create a more accepting and encouraging atmosphere for females who are infertile. Stakeholders may improve the mental and emotional health of females who are stigmatized for infertility by addressing these elements, which will ultimately improve their quality of life.

## Data Availability

The datasets presented in this study can be found in online repositories. The names of the repository/repositories and accession number(s) can be found in the article/Supplementary Material.

## Appendix

**Consent to Participate**

**To Whom It May Concern,**

I, ———————————————-, confirm that I have read and understood the information provided regarding the research study titled “Do Perceived Social Support Mitigate the Influence of Infertility Stigma on Fertility Quality of Life?”. I acknowledge that my participation in this study is entirely voluntary.

I understand that:

1. My participation is confidential, and my personal information will not be disclosed.
2. I have the right to withdraw from the study at any time without facing any penalties.
3. I have had the opportunity to ask questions about the study and have received satisfactory answers.
4. I hereby give my consent for the publication of the findings derived from my participation in this research, with the assurance that my identity and personal information will remain confidential.

By signing below, I give my informed consent to participate in this research study.

**Participant’s Name**: ___________________________

**Signature**: ___________________________

**Date**: ___________________________

**Researcher’s Name**: ___________________________

**Signature**: _______________________

-----------------------------
